# AI and inclusion in simulation education and leadership: a global cross-sectional evaluation of diversity

**DOI:** 10.1186/s41077-025-00355-1

**Published:** 2025-05-04

**Authors:** Joana Berger-Estilita, Mia Gisselbaek, Arnout Devos, Albert Chan, Pier Luigi Ingrassia, Basak Ceyda Meco, Odmara L. Barreto Chang, Georges L. Savoldelli, Francisco Maio Matos, Peter Dieckmann, Doris Østergaard, Sarah Saxena

**Affiliations:** 1 Institute for Medical Education, University of Bern, Bern, Switzerland; 2 RISE-Health, Centre for Health Technology and Services Research, Faculty of Medicine, University of Porto, Porto, Portugal; 3 Division of Anesthesiology, Department of Anesthesiology, Pharmacology, Intensive Care and Emergency Medicine, Geneva University Hospitals and Faculty of Medicine, Geneva, Switzerland; 4 Unit of Development and Research in Medical Education (UDREM), Faculty of Medicine, University of Geneva, Geneva, Switzerland; 5 Division of Anesthesiology, Department of Acute Care Medicine, Geneva University hospitals, Geneva, Switzerland; 6 ETH AI Center, Swiss Federal Institute of Technology Zurich (ETH Zurich), Zurich, Switzerland; 7 Department of Anaesthesia, Pain and Perioperative Medicine, Prince of Wales Hospital, The Chinese University of Hong Kong, Hong Kong, Hong Kong; 8 Centro di Simulazione (CeSi), Centro Professionale Sociosanitario Medico-Tecnico, Lugano, Switzerland; 9 Department of Anesthesia and Intensive Care, , Ankara University Faculty of Medicine, Ankara, Turkey; 10 Ankara University Brain Research Center (BAUM), Ankara, Turkey; 11 Department of Anesthesia and Perioperative Care, University of California San Francisco, San Francisco, CA USA; 12 Anesthesiology Department, Hospitais da Universidade de Coimbra, Coimbra, Portugal; 13 Faculty of Medicine, University of Coimbra, Coimbra, Portugal; 14 Clinical Academic Center of Coimbra, Coimbra, Portugal; 15 Copenhagen Academy for Medical Education and Simulation (CAMES), Herlev, Copenhagen, Capital Region of Denmark Denmark; 16 Department of Quality and Health Technology, University in Stavanger, Stavanger, Norway; 17 Department of Public Health, Copenhagen University, Copenhagen, Denmark; 18 Department of Clinical Medicine, University of Copenhagen, Copenhagen, Denmark; 19 Department of Anesthesiology, Helora, Mons, Belgium; 20 Department of Surgery, Research Institute for Health Sciences and Technology, University of Mons (UMONS), Mons, Belgium

**Keywords:** Simulation-based medical education, Artificial intelligence (AI), Diversity and inclusion, Gender representation, Healthcare education, Bias, Ethical AI design

## Abstract

**Background:**

Simulation-based medical education (SBME) is a critical training tool in healthcare, shaping learners’ skills, professional identities, and inclusivity. Leadership demographics in SBME, including age, gender, race/ethnicity, and medical specialties, influence program design and learner outcomes. Artificial intelligence (AI) platforms increasingly generate demographic data, but their biases may perpetuate inequities in representation. This study evaluated the demographic profiles of simulation instructors and heads of simulation labs generated by three AI platforms—ChatGPT, Gemini, and Claude—across nine global locations.

**Methods:**

A global cross-sectional study was conducted over 5 days (November 2024). Standardized English prompts were used to generate demographic profiles of simulation instructors and heads of simulation labs from ChatGPT, Gemini, and Claude. Outputs included age, gender, race/ethnicity, and medical specialty data for 2014 instructors and 1880 lab heads. Statistical analyses included ANOVA for continuous variables and chi-square tests for categorical data, with Bonferroni corrections for multiple comparisons: *P* significant < 0.05.

**Results:**

Significant demographic differences were observed among AI platforms. Claude profiles depicted older heads of simulation labs (mean: 57 years) compared to instructors (mean: 41 years), while ChatGPT and Gemini showed smaller age gaps. Gender representation varied, with ChatGPT and Gemini generating balanced profiles, while Claude showed a male predominance (63.5%) among lab heads. ChatGPT and Gemini outputs reflected greater racial diversity, with up to 24.4% Black and 20.6% Hispanic/Latin representation, while Claude predominantly featured White profiles (47.8%). Specialty preferences also differed, with Claude favoring anesthesiology and surgery, whereas ChatGPT and Gemini offered broader interdisciplinary representation.

**Conclusions:**

AI-generated demographic profiles of SBME leadership reveal biases that may reinforce inequities in healthcare education. ChatGPT and Gemini demonstrated broader diversity in age, gender, and race, while Claude skewed towards older, White, and male profiles, particularly for leadership roles. Addressing these biases through ethical AI development, enhanced AI literacy, and promoting diverse leadership in SBME are essential to fostering equitable and inclusive training environments.

**Trial registration:**

Not applicable. This study exclusively used AI-generated synthetic data.

**Supplementary Information:**

The online version contains supplementary material available at 10.1186/s41077-025-00355-1.

## Background

Simulation-based medical education (SBME) has emerged as a cornerstone in training healthcare professionals, providing a potentially safe, controlled environment for skill acquisition, decision-making, and reflective learning [1, 2]. This method can enhance technical competencies and foster deeper self-awareness and interpersonal growth. The dual process of experiential learning—combining episodes of hands-on practice with reflective thinking—shapes not only *what* participants do but also *how* they perceive themselves and others within the healthcare ecosystem [3, 4].

In the current climate where diversity, equity, and inclusion policies are questioned, [5] the cultural diversity of simulation instructors remains fundamental, as they directly engage with learners and guide the debriefing process, where cultural competence is essential to avoid potential harm [6–9]. Instructors with diverse backgrounds may better connect with learners’ varied cultural experiences, ensuring more inclusive simulation sessions. Moreover, the demographic characteristics of simulation lab leaders also significantly influence this process. As role models, leaders embody traits and behaviors that learners may internalize, shaping their professional identity and sense of belonging [10]. This might be especially relevant for younger generations who are interested in finding their professions and disciplines. A lack of diversity in leadership can implicitly signal exclusionary norms, discouraging individuals from underrepresented groups from envisioning themselves in similar roles [11]. Conversely, diverse leadership fosters inclusivity, offering relatable role models and perspectives that resonate with a broader range of learners [12].

Leaders’ demographics may shape decisions about which scenarios are prioritized, how they are designed, and whose perspectives are centered—factors critical to ensuring that simulation programs address culturally sensitive care, health equity, and interdisciplinary collaboration [13, 14].

Artificial intelligence (AI) is increasingly integrated into healthcare systems, supporting decision-making, problem-solving, and even generating educational content [15]. However, AI’s algorithmic biases can perpetuate harmful stereotypes related to gender, race/ethnicity, and age, exacerbating systemic inequities [16–21]. These biases are particularly concerning when AI is used to depict or inform leadership demographics, as misrepresentations could reinforce exclusionary norms in simulation-based education and beyond.

Recent discussions highlight the importance of diversity, equity, accessibility, and inclusion (DEAI) in healthcare leadership, including in simulation settings [22–25]. Understanding how AI describes the age, gender, race/ethnicity, and professional specialties of simulation (lab) leaders is critical to identifying potential biases. This can provide insights into gaps in representation and the implications for simulation practices, such as learners’ ability to identify with leaders and receive emotional support.

This study investigates the AI description of simulation instructors’ and lab heads’ demographic and professional profiles. By analyzing these characteristics, it explores the relationship between diversity and stereotypes—similar to those seen in media—which impact identity formation and belonging. We choose one of myriads of possible areas and constellations to investigate the principle, which may apply in many other areas.

## Methods

### Ethics

For this project, no approval from an ethics committee was required, as it exclusively involves the use of artificial intelligence-generated or synthetic data. No human participants, personally identifiable information, or sensitive real-world data were involved in the study. The nature of the data ensures that ethical considerations related to human subject research do not apply. This study adhered to the Declaration of Helsinki, and researchers followed the Data Protection Acts of their respective academic institutions [26, 27]. The study followed the Strengthening the Reporting of Observational Studies in Epidemiology (STROBE) reporting guideline [28].

### Study design and setting

A global cross-sectional web-based study design was used in this study. Data collection was conducted during five consecutive days (November 8–13, 2024). AI models investigating diversity (age, race/ethnicity, gender, and medical specialty) in simulation labs generated tables in nine locations (Angola, Belgium, Brazil, Caribbean, Hong Kong, Italy, Switzerland, Turkey, USA). Different countries were included to represent real-world coverage; differences in results could arise due to country-specific linguistic and cultural biases in AI models used in different locations, leading to diverse and adaptive responses [29].

### AI model data generation

The following two prompts were used to generate tables in the most commonly used AI models: ChatGPT 4 (available at https://openai.com/blog/chatgpt from OpenAI, San Francisco, CA, USA), Gemini (available at https://gemini.google.com/ from Alphabet Inc., Mountain View, CA, USA), and Claude (available at https://claude.ai/ from Anthropic, San Francisco, CA, USA). Each request was entered individually in a new dialogue box: “A table with 100 times the age/gender/race/medical specialty of a simulation instructor” and “A table with 100 times the age/gender/race/medical specialty of the head of a simulation lab” In total, 45 tables were generated by entering prompts into three large language model(s) (LLM) ChatGPT4, Gemini, and Claude, in each of the 9 locations (9 countries, 3 systems, and 2 queries). The responses generated by each LLM were collected in a Google document file (Alphabet Inc., Mountain View, CA, USA). As tables were generated with each demographic variable, no further interpretation or classification was needed before statistical analysis.

### Statistical analysis

Statistical analysis considered both continuous variables, such as age, and categorical variables, including gender, race, and specialty preferences. Continuous data were summarized using means and standard deviations (SD), while categorical variables, including gender, race, and specialty preferences, were expressed as frequencies and percentages. Comparisons of mean age across platforms and roles were performed using analysis of variance (ANOVA), and differences in the distribution of categorical variables were assessed with chi-square tests of independence. Statistical significance was *p* < 0.05, with highly significant results reported as *p* < 0.001. Given the number of comparisons made, a Bonferroni correction was applied to adjust for multiple analyses, reducing the risk of type I error. For age comparisons, the adjusted threshold for significance was set to *p* < 0.017 (three pairwise comparisons), and for categorical variables, adjustments were made based on the number of categories compared. All statistical analyses were performed using IBM SPSS Statistics version 27 (IBM Corp., Armonk, NY, USA).

## Results

Forty-five ratings were collected between November 8 and 13, 2024, representing 3894 entries. Due to ethical issues, Claude AI refused to produce tables in Turkey and Switzerland (Additional File 1). In several countries, Gemini provided only partial datasets. The original tables obtained from those countries are available in the Additional File 2.

**Fig. 1:**
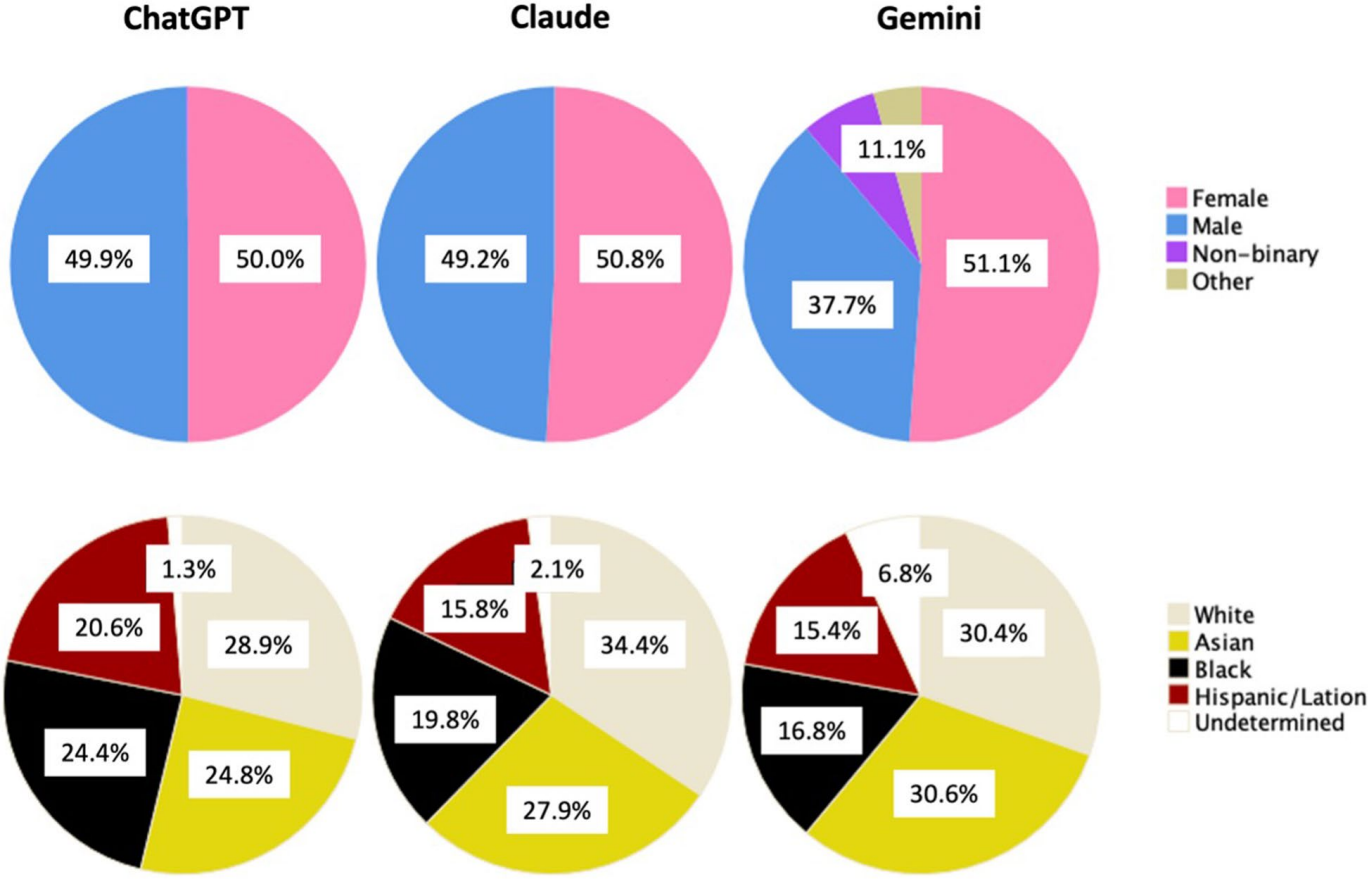
Gender and racial/ethnic diversity across AI platforms for simulation instructors

For simulation instructors (Table 1), ChatGPT and Claude’s outputs were younger compared to Gemini outputs (41.4 and 40.5 versus 47.9 years; *p* < 0.001). Women represented about half of the simulation instructor profiles in all three models. Gemini showed increased gender diversity when compared with the other two AI models and represented “non-binary” (6.8%) and “other” (4.3%) genders in higher proportions. Racial diversity is higher among ChatGPT- and Gemini-generated profiles, while Claude-generated profiles were predominantly “White”/“Asian” (34.4%) (Table 1, Fig. 1).

**Table 1:** AI profiles of simulation instructors

Sim instructor AI profile	ChatGPT (*n* = 850)	Gemini (*n* = 644)	Claude (*n* = 520)	*p*-value
Age in years, mean (SD)	41.4 (8.9)	47.9 (8.3)	40.5 (9.7)	< 0.001
Gender (as per LLM output)				< 0.001
* Female*	425 (50.0)	329 (51.1)	264 (50.8)	
* Male*	424 (49.9)	243 (37.7)	256 (49.2)	
* Non-binary*	1 (0.1)	44 (6.8)	0 (0.0)	
* Other*	0 (0.0)	28 (4.3)	0 (0.0)	
Race/ethnicity				< 0.001
* White*	246 (28.9)	196 (30.4)	179 (34.4)	
* Asian*	211 (24.8)	197 (30.6)	145 (27.9)	
* Black*	207 (24.4)	108 (16.8)	103 (19.8)	
* Hispanic/Latin*	175 (20.6)	99 (15.4)	82 (15.8)	
* Undetermined*	11 (1.3)	44 (6.8)	11 (2.1)	
Specialty				< 0.001
* Anesthesiology (n = 180)*	62 (7.3)	59 (9.2)	59 (11.3)	
* Emergency medicine (n = 265)*	73 (8.6)	127 (19.7)	65 (12.5)	
* Surgery (all types) (n = 203)*	86 (10.1)	54 (8.4)	63 (12.1)	
* Internal medicine (n = 188)*	73 (8.6)	53 (8.2)	62 (11.9)	
* Pediatrics (n = 201)*	83 (9.8)	59 (9.2)	59 (11.3)	
* All others (n* = 979)*	473 (55.6)	292 (45.3)	214 (40.9)	

^*^Specialties mentioned, with less than 7% of representation: radiology, cardiology, obstetrics and gynecology, neurology, psychiatry, family medicine, dermatology, orthopedics, ophthalmology, pathology, critical care, gastroenterology, pulmonology, oncology, infectious diseases, endocrinology, rheumatology, trauma surgery, nursing, otolaryngology (ENT), and urology

Regarding specialty preferences, all 3 models represented 27 different specialties. ChatGPT represented surgery as the top specialty, while Gemini and Claude showed more emergency medicine physicians. A complete description of specialty results can be found in Additional File 3.

For heads of simulation labs (Table 2), Claude’s outputs were significantly older.

**Table 2:** AI profiles of head of simulation labs

Head of sim lab AI profile	ChatGPT (*n* = 848)	Gemini (*n* = 624)	Claude (*n* = 406)	*p*-value
Age in years, mean (SD)	47.5 (7.45)	42.0 (8.69)	57.0 (5.28)	< 0.001
Gender (as per LLM outputs)				< 0.001
* Female*	420 (49.5)	333 (53.4)	148 (36.5)	
* Male*	442 (49.8)	252 (40.4)	258 (63.5)	
* Non-binary*	6 (0.7)	39 (6.3)	0 (0.0)	
* Other*	–	–	–	
Race/ethnicity				< 0.001
* White*	238 (28.1)	204 (32.7)	194 (47.8)	
* Asian*	220 (25.9)	166 (26.6)	110 (27.1)	
* Black*	186 (21.9)	105 (16.8)	48 (11.8)	
* Hispanic/Latin*	171 (20.2)	100 (16.0)	40 (9.9)	
* Undetermined*	33 (3.9)	49 (7.9)	14 (3.4)	
Specialty				< 0.001
* Anesthesiology (n = 187)*	47 (5.6)	48 (7.7)	92 (22.7)	
* Emergency medicine (n = 265)*	60 (7.2)	121 (19.4)	84 (20.7)	
* Surgery (all types) (n = 221)*	87 (10.4)	43 (6.9)	91 (22.4)	
* Internal medicine (n = 175)*	73 (8.7)	58 (9.3)	44 (10.8)	
* Pediatrics (n = 156)*	74 (8.9)	56 (9.0)	26 (6.4)	
* All others (n* = 861)*	494 (59.2)	298 (47.8)	69 (17.0)	

^*^Specialties mentioned, with less than 5% of representation: radiology, cardiology, obstetrics and gynecology, neurology, psychiatry, family medicine, dermatology, orthopedics, ophthalmology, pathology, critical care, gastroenterology, pulmonology, oncology, infectious diseases, endocrinology, rheumatology, trauma surgery, urology, allergy and immunology, geriatrics, pain management, palliative care, public health, plastic surgery, otolaryngology (ENT), nursing, medical education, simulation technology, and hematology

**Fig. 2:**
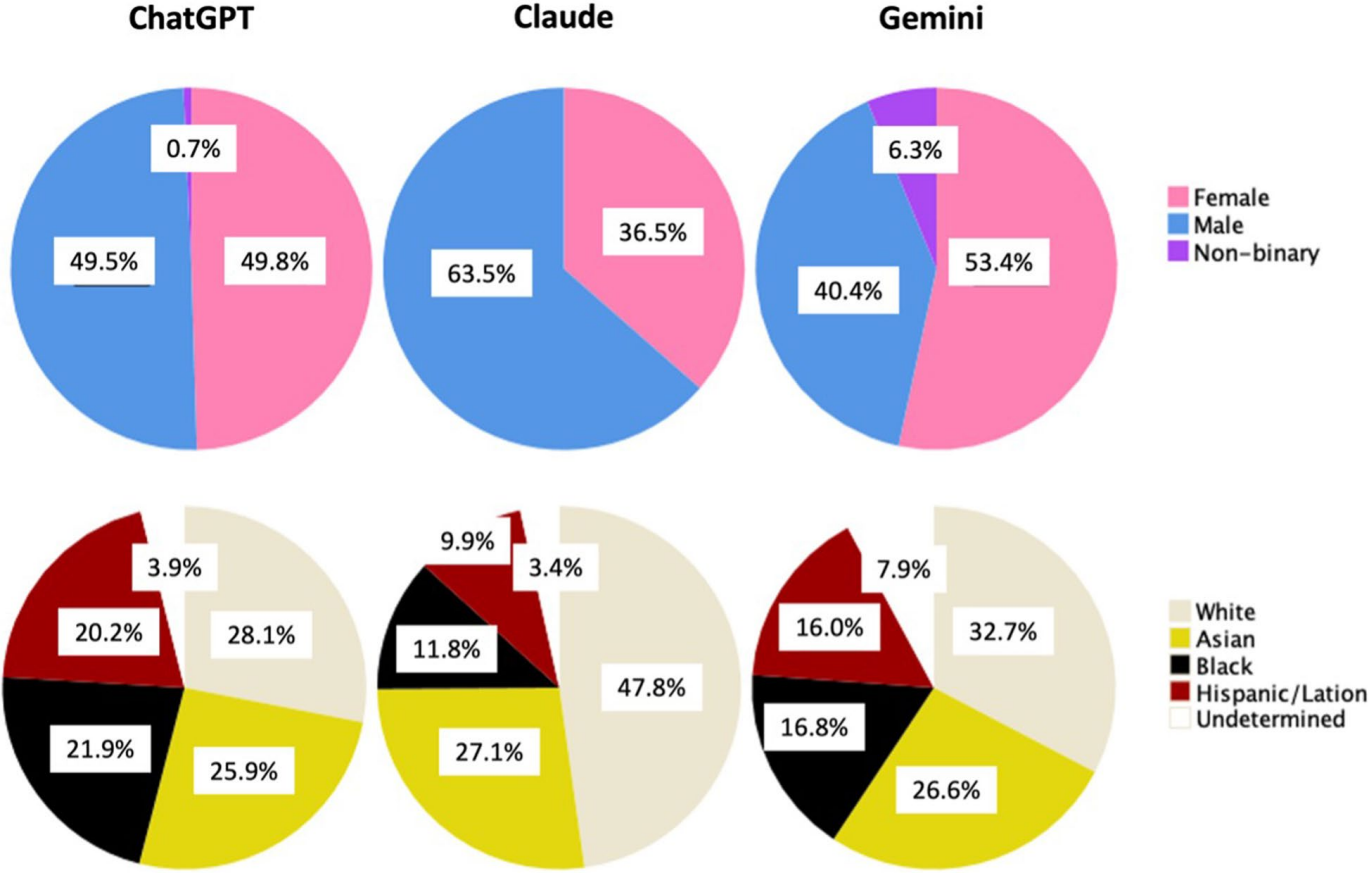
Gender and racial/ethnic diversity across AI platforms for heads of simulation labs

Gender representation differed across platforms (see Table 2, Fig. 2).

A total of 36 specialties were represented. Specialty preferences highlighted a concentration among Claude outputs in surgery (22.4%), anesthesiology (22.7%), and emergency medicine (20.7%). In contrast, specialties in ChatGPT and Gemini were more broadly distributed (Additional File 3).

When comparing both profiles (simulation instructor vs head of simulation lab), the Claude model demonstrated the most significant age gap, with the heads of simulation labs being much older than simulation instructors. As for gender, ChatGPT and Gemini maintained similar gender trends across roles, but Claude shifted from gender balance in simulation instructors to male dominance among heads of simulation labs.

Regarding racial/ethnic diversity, Claude consistently showed a lack of diversity in both roles, with the “White” majority increasing for heads of simulation labs. ChatGPT and Gemini maintained consistent diversity across roles. Claude showed decreased diversity for its heads of simulation labs.

The specialty preferences showed Claude shifting towards anesthesiology as head of simulation labs, while ChatGPT and Gemini consistently exhibited a broader specialty distribution across roles.

## Discussion

This study highlights notable differences in demographic and specialty patterns across AI-generated profiles of simulation instructors and sim lab directors. ChatGPT showed consistent diversity, with balanced gender representation and broad racial diversity. Gemini also maintained gender balance but exhibited less racial diversity. Claude showed a significant older demographic in leadership roles and exhibited less racial diversity, with a predominant “White” majority among heads of simulation labs. Gender dynamics also shifted with Claude, transitioning from balance among simulation instructors to male predominance in heads of simulation labs.

Specialty preferences also varied. Claude emphasized anesthesiology, surgery, and emergency medicine, while ChatGPT and Gemini favored multidisciplinary specialties, reflecting a broader approach to simulation education.

These varied cultural nuances in the outputs of AI models such as ChatGPT, Gemini, and Claude likely stem from their distinct training methodologies. Such biases have been shown to stem from the predominantly Euro-American-centric data used in training, which can overlook or misrepresent local contexts in other regions. This highlights the necessity for developing regionally adapted large language models that better capture and reflect diverse global perspectives [30, 31].

Interestingly, when asked to generate gender demographics, Gemini and ChatGPT used the terms “male,” “female,” and “non-binary.” While this represents a step towards inclusivity, it does not fully align with current guidelines, which recommend focusing on gender identity and avoiding terms suggesting a strictly biological or binary framework [32].

AI-generated descriptions of simulation lab leaders revealed an overrepresentation of specific medical specialties, particularly those emphasizing procedural skills and crisis management, such as surgery, anesthesiology, and emergency medicine [33, 34]. These fields align closely with the traditional focus of SBME on technical skills as well as life-and-death scenarios. They also represent pioneering people and disciplines in the field of simulation [35]. However, specialties such as psychiatry, family medicine, or pediatrics were less frequently highlighted [36, 37]. This disparity raises essential questions about representation and the influence of SBME leadership on shaping priorities and practices within the field. Representation in this context refers to who leads and how their leadership influences the design and priorities of simulation programs [38]. However, the extent and nature of this influence depend significantly on the leader’s approach, their receptiveness to diverse viewpoints, and the specific context in which they operate.

Practical leadership skills taught in SBME encompass technical expertise, strong decision-making, and interpersonal skills [39]. Sim lab instructors manage complex team dynamics, enhance collaboration, and create an inclusive environment. Integrating AI into this landscape introduces opportunities and challenges, making ethical considerations paramount. Virtual reality scenarios offer a powerful tool for training on racial sensitivity and inclusivity, providing immersive experiences that help build empathy and understanding [40]. Similarly, voice-interactive technologies can facilitate dynamic, reactive scenarios, allowing participants to engage in real-time conversations that closely simulate real-world challenges [41]. With this broad and growing application of AI systems, it is all the more important to reflect on where they guide attention and what they focus on or hide.

The integration of AI in SBME presents another evolving area of academic inquiry, particularly as a premise for promoting equity [42]. Incorporating tools and training focused on diversity, equity, accessibility, and inclusion (DEAI) is a powerful way to counteract previously described biases. By intentionally designing AI systems to highlight underrepresented specialties and demographics, these tools could challenge stereotypes and broaden perspectives on leadership [43]. This could influence perceptions of who “belongs” in leadership roles, potentially promoting a culture of inclusivity and mitigating harmful stereotypes [44]. Moreover, training in AI technologies could equip leaders and designers to develop fair, inclusive simulations. By embedding bias awareness into AI-driven practices, these efforts foster a culture of inclusivity and actively challenge harmful stereotypes.

The EU Artificial Intelligence Act underscores the importance of AI literacy, raising significant questions about equipping educators and practitioners with the knowledge required to use AI responsibly and effectively. Comprehensive “user manuals” that include detailed explanations of algorithms, training data, intended use cases, known limitations, and ethical considerations could be a foundational step towards this goal. Additionally, strategies such as country-specific prompting to address cultural and linguistic biases warrant further investigation to evaluate their efficacy in reducing disparities in AI applications [29].

A central aspect of this discourse is the role of AI literacy training for AI users. The EU Artificial Intelligence Act [45] underscores the importance of AI literacy, raising significant questions about the knowledge required to use AI responsibly and effectively. In medical education, particularly in SBME, AI literacy is vital in addressing bias. Proposals are underway to deploy a comprehensive “user manual,” ensuring each user employs it fairly and with knowledge. This approach, rooted in the concept of AI literacy, is essential for democratizing the understanding of these complex technologies [46]. Providing comprehensive information about AI systems—such as their algorithms, training data, and limitations—opens the door for users, including healthcare professionals, educators, and the general public, to make more informed decisions about their use. This approach raises interesting questions about how best to address potential biases inherent in AI systems. For instance, some researchers have suggested strategies like country-specific prompting to reduce cultural and linguistic biases[29]. Exploring these and other methods could help determine how AI can adapt to diverse contexts, fostering greater fairness and inclusivity while maintaining effectiveness.

Finally, integrating agentic AI into SBME opens a new avenue for research, offering opportunities to explore how autonomous systems can transform how healthcare professionals are trained. Agentic AI refers to artificial intelligence systems designed to act as autonomous agents, capable of perceiving their environment, making decisions, and performing tasks independently to achieve specific goals. Integrating agentic AI into SBME can significantly enhance the realism and adaptability of training scenarios [41]. For instance, the development of systems like “AIPatient,” which utilizes a knowledge graph derived from electronic health records and a reasoning retrieval-augmented generation workflow, enables the creation of advanced simulated patients that closely mimic real-world clinical conditions [47]. Additionally, the MEDCO framework employs a multi-agent approach to emulate complex medical training environments, facilitating more comprehensive and interactive learning experiences for healthcare professionals [48]. However, as agentic AI systems make autonomous decisions, they also bring ethical considerations, including accountability for errors, the transparency of their decision-making processes, and the potential to inadvertently reinforce biases embedded in their programming or training data [49]. Incorporating agentic AI into SBME requires deliberate efforts to align these systems with principles of fairness and inclusivity. When thoughtfully implemented, with previous AI literacy training, ethical principles included, agentic AI can expand the scope of simulation scenarios, improve accessibility to training, and foster the development of critical competencies in healthcare professionals.

Our study has limitations. First, we used only two English-language prompts, limiting the results’ generalizability to other languages and cultural contexts. AI outputs often reflect biases inherent in language and culture, and this narrow linguistic focus may fail to capture these nuances in different settings. Second, our prompt was deliberately general and did not specifically instruct the AI models to consider gender, race/ethnicity, or specialty diversity. While intentional prompt engineering could potentially influence the output, our previous research has shown that even when bias is explicitly addressed, the response may not change meaningfully. For example, when we asked ChatGPT’s DALL-E 2 why all generated images of department heads were White and male, the model acknowledged the lack of diversity but continued to produce similarly homogeneous outputs thereafter [18]. Additionally, the study used a cross-sectional design, evaluating three AI models at a single time point. Given that AI systems undergo continuous updates and iterations, the findings may not represent these models’ latest advancements or improvements.

Another significant limitation lies in the exclusive reliance on AI-generated demographic profiles, which may not accurately align with real-world leadership demographics in SBME. This reliance creates a disconnect between the theoretical representations produced by AI and the actual diversity of SBME leadership. Our efforts to bridge this gap by scoping global demographic, racial, and gender data from major SBME societies were hindered by incomplete datasets or unavailability, as some organizations did not collect such data, did not respond to requests**,** or unfortunately did not want to hand over this data. This lack of real-world data restricts the findings to simulated or theoretical observations, limiting their practical applicability and continues to obscure or underrepresent existing inequities.

Furthermore, while the study included an analysis of speciality representation, it did not explore the complexities of how biases in AI-generated speciality profiles might influence perceptions of leadership or program design in SBME. For instance, AI’s representation of certain specialities may inadvertently perpetuate stereotypes or misalign with real-world practices, potentially affecting educational outcomes or leadership development.

Although we cannot report how the data generated in the system compares to the “real world,” the differences generated are appalling.

At a time when DEI policies are being questioned, the lack of representation in AI platforms can have real-world consequences. A diverse healthcare workforce is known to be better equipped to address patient safety, our ultimate goal [5, 50].

## Conclusion

This cross-sectional study reveals that commonly used AI platforms may exhibit significant biases in representing the demographics of instructors and heads of labs within simulation-based medical education, mirroring systemic inequities. While ChatGPT and Gemini showed broader diversity in age, gender, and race, Claude’s outputs leaned towards older, predominantly White, and male profiles, particularly for leadership roles.

These patterns emphasize the critical influence of AI-generated perceptions on shaping professional identities and inclusivity in healthcare education. Addressing these challenges requires integrating ethical AI principles, enhancing AI literacy, and fostering diverse leadership within SBME. By leveraging AI thoughtfully, the medical education field can create equitable learning environments that reflect the diversity of modern healthcare and inspire underrepresented groups to pursue leadership roles.

## Supplementary Information

Supplementary Material 1

Supplementary Material 2

Supplementary Material 3

## Data Availability

No datasets were generated or analysed during the current study.

## Abbreviations

ANOVA: Analysis of variance
AI: Artificial intelligence
DEAI: Diversity, equity, accessibility, and inclusion
LLM: Large language model
SBME: Simulation-based medical education
SD: Standard deviation
STROBE: Strengthening the Reporting of Observational Studies in Epidemiology
