# Supplementary figures and images for: AI and inclusion in simulation education and leadership: a global cross-sectional evaluation of diversity
Source: Adv Simul (Lond). 2025 May 4;10:26. doi: 10.1186/s41077-025-00355-1 (PMC12049791; doi:10.1186/s41077-025-00355-1)

Additional File 3: Supplementary statistics

Supplemental 3

Sim Instructor


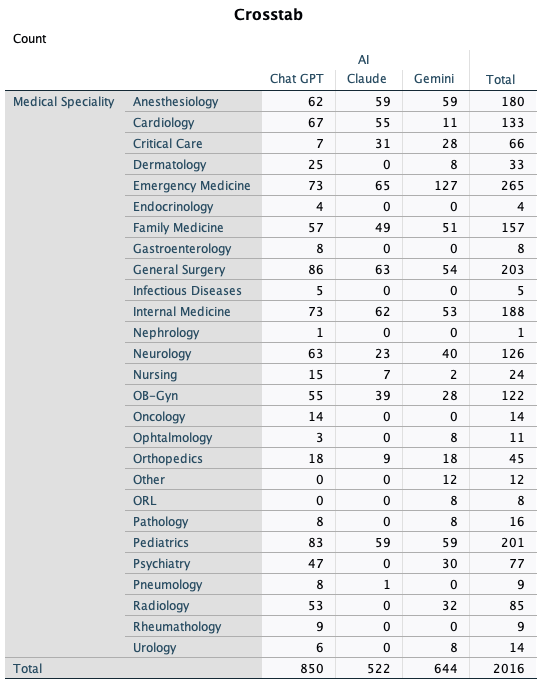


Sim Head of Lab


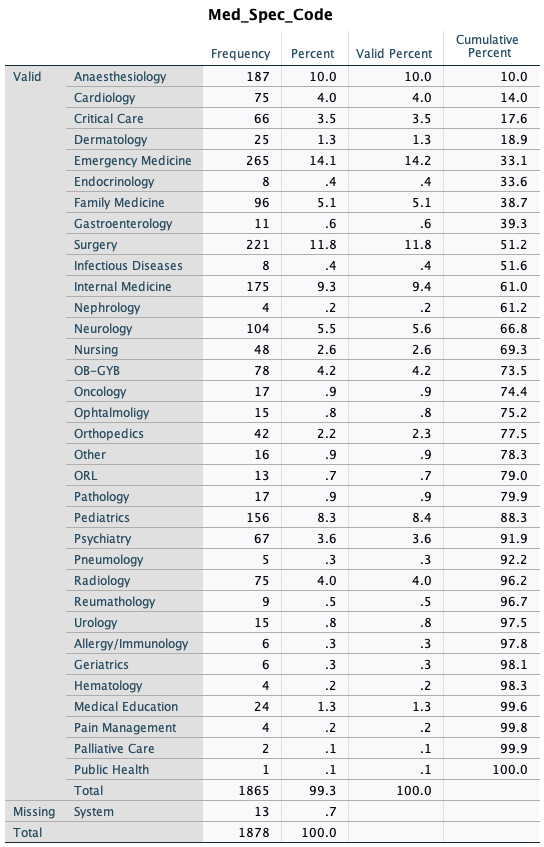


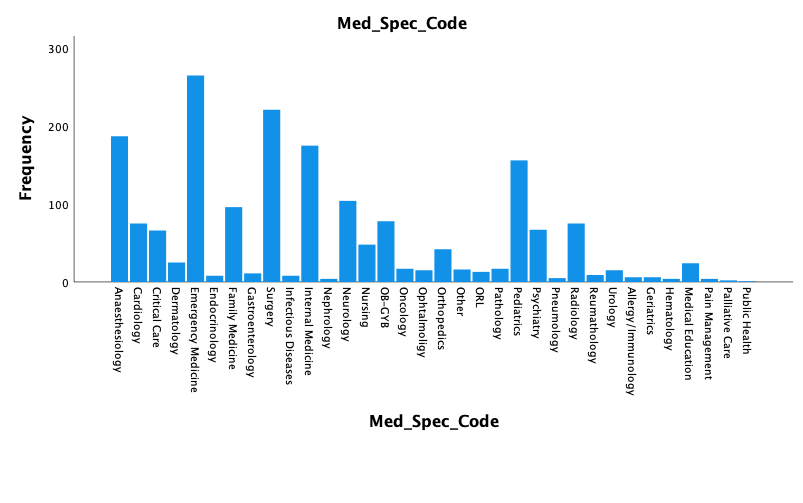

Supplement: Supplementary file 3 — Supplementary Material 3 [file 41077_2025_355_MOESM3_ESM.docx]
